# Design and Primary Investigations of a Double Ring Loop Antenna for Ice, Frost and Wildfire Detection in Early Warning Systems

**DOI:** 10.3390/s26010155

**Published:** 2025-12-25

**Authors:** Rula Alrawashdeh

**Affiliations:** Electrical Engineering Department, Mutah University, Al-Karak 61710, Jordan; rular18@mutah.edu.jo

**Keywords:** early warning systems, frost, ice, sensitivity, sensor, water, wildfire

## Abstract

In this paper, a flexible rectangular loop antenna is designed and proposed for ice, frost and wildfire detection. The antenna is composed of two concentric rings made of a flexible conductor. The proposed antenna was responsive to different materials based on distinct shifts in the resonant frequency, which was employed to differentiate between these materials. The antenna provides a wide response and sensitivity range to detect ice or frost with relative permittivity close to 3 and water with relative permittivity close to 72 at the same time. This wide sensitivity level is attributed to the internal loop which works with the external ring to form a capacitor with a capacitance varying with the relative permittivity of the material under test. The internal loop also enhances coupling with the material under test and fine-tunes the antenna’s response. The antenna achieved a maximum radiation efficiency of 97.1% and gain of 2.83 dBi at 2.45 GHz across the tested scenarios involving frost and ice. It also obtains a maximum radiation efficiency and gain of up to 6.67% and −8.27 dBi, respectively, for water at 40 °C and 50 °C, respectively. Additionally, the antenna preserves the same direction of maximum radiation for all of the investigated materials, which minimizes constraints on the receiving antenna’s radiation pattern requirements. The proposed antenna features simplicity, robust performance and a wide sensitivity range over temperatures between 0 and 50 °C, which makes it a good candidate for environmental monitoring.

## 1. Introduction

In addition to data transmission, antennas have been recently used as sensors for applications such as early warning and environmental monitoring. In such systems, the antenna is employed to detect natural disasters or to sense an environmental parameter and send a corresponding alarm signal to an external receiver. This reduces the hardware and power consumption needed if sensors separate from antennas are used [1,2,3,4,5]. In addition, it allows accurate and rapid notification.

Antennas in general are usually required to satisfy a set of specifications including sufficient gain, robust performance and good impedance matching. They can be made of a single element or arranged in an array of multiple elements. While antenna arrays may boost the gain, improve the radiation pattern and help in interference cancellation [6], they increase the system complexity. Sensor antennas need to meet further requirements such as the ability to detect slight variations in their surrounding environment. They are also required to distinguish between different materials with very similar dielectric properties.

These requirements, in addition to compactness and ease of integration with existing system components, need accurate design considerations involving the following:

- Specific miniaturization techniques that should maintain good inclusion of the sensing elements within the structure. Inclusion of split rings and complementary split rings could be a good candidate in this case [7,8].
- Effective near-electromagnetic field control. As a sensor, the antenna needs to couple with the material under test [9]. Again, structures based on rings and loops can work to improve the capacitive electric and magnetic field coupling, which plays a major role in improving sensitivity [10].

In systems such as early warning systems, the sensor antenna is also required to maintain robust performance under harsh conditions accompanying natural disasters [11]. Specific examples include frost damage and wildfire, which can be detected by the antenna at an early stage before their occurrence.

**Figure 1:**
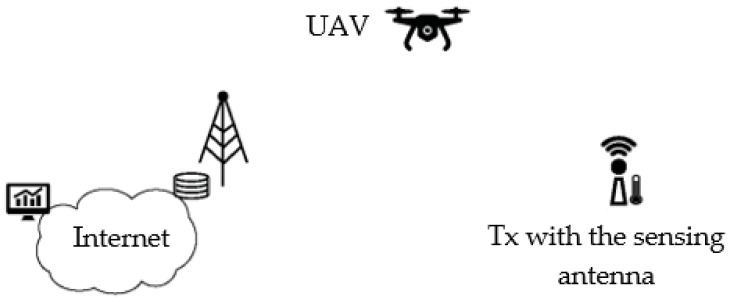
A typical wireless natural disaster early warning system.

An example of an early warning system is depicted in Figure 1, where the antenna detects the upcoming disaster such as frost or wildfire and transmits the alarm data to an external receiver, which could be a fixed base station or a mobile autonomous aerial vehicle (UAV). Hence, in such a system the sensor antenna must fulfill additional design requirements such as an omnidirectional radiation pattern [12] to allow data transmission for both the fixed base station and the mobile UAV at the same time.

Various sensor antennas have been proposed in the literature for various applications. The antenna in [4] was used for sensing strain and humidity; a dipole antenna was designed based on inkjet printing of a copper layer on a Polyimide film. Sensing was obtained by measuring the resonant frequency shift in response to the antenna stretching under different humidity and strain levels. In [13], a closed S-shaped monopole antenna was presented as an implantable sensor for detecting breast tumors. Detection of the surrounding tissue composition was enabled by analyzing its resonance frequency. A flexible, wearable patch antenna was introduced in [14] for pressure sensing, featuring a resonant frequency of 4.8 GHz and a frequency shift range of 8% under different strain conditions. Another wearable sensor antenna was presented in [15]. It was a textile monopole capable of breast cancer detection and monitoring over the 1.8 to 10 GHz ISM band. Different parameters, including the resonant frequency, matching level and transmission coefficient, were used for detecting the tumor’s existence and size. Antennas designed for detecting material compositions have also been investigated, with examples provided in [16,17]. The same concept of varying the antenna’s resonant frequency in response to variations in the dielectric permittivity of the material under test was employed.

Sensor antennas have been also proposed for early warning systems and environmental monitoring. The most common type is the patch antenna, which can accommodate various slots and structures that facilitate coupling. In [18], a T-slot antenna was proposed for ice and frost detection at 2.45 GHz, while another patch antenna with a cross slot was introduced in [1] for sensing at 5.6 GHz. A substrate-integrated waveguide (SIW) antenna was also developed in [19] and presented for ice, frost and wildfire detection at 5.6 GHz. Although the design incorporated smart sensing capabilities supported by Machine Learning (ML), it featured several holes with small radii and short spacing, making it complex to fabricate. The study in [20] compared two antenna designs, a loop and a dipole, demonstrating that the loop antenna exhibited higher sensitivity than the dipole antenna.

Loop and ring antennas in general feature many characteristics, including ease of integration and conformity to many designs and structures. They can be in different shapes, such as circular or rectangular, or they can even fit to specific conformal structures [21]. The loop antenna can also obtain different polarization types such as circular polarization, which is appealing for different applications [22]. It can host split rings and complementary split rings, which provide a set of appealing features as discussed above [23]. Moreover, it can be optimized to control near electromagnetic fields [24].

All of these appealing features proved by the promising performance of the loop antenna in [20] inspired the investigations and design presented in this work, which seeks to further study and investigate loop antennas for sensing.

This work utilizes an internal rectangular loop coupled with an external feeding loop to control the area of coupling with the material under test (MUT). This results in adjusting the sensitivity level accordingly. The proposed antenna can detect different materials resembling ice, frost and water at 40 °C and 50 °C, mimicking wildfire conditions. The overall antenna performance for both sensing and data transmission is analyzed, evaluated and validated. The antenna proposed in this work is likely the first loop antenna with demonstrated detection capabilities for ice, frost and wildfire simultaneously.

This paper is organized as follows:

First, the design structure and concept are presented in Section 2. Next, the methodology and methods employed in this research are outlined in Section 3. The scenarios for both data sensing and transmission are then simulated and discussed and analyzed in Section 4, which also includes link budget calculations. Simulation results relevant to ice are then validated through measurements in Section 5. Finally, the paper is concluded in the last section.

## 2. Structures and Concept

**Figure 2:**
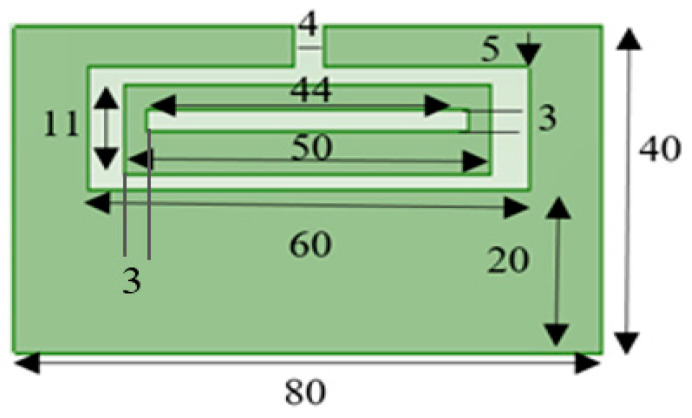
The proposed antenna structure; dimensions in mm.

The proposed antenna is composed of two rectangular loops coupled to each other. It is made of a flexible conductor (copper tape). The antenna structure is shown in Figure 2.

The structure and materials were selected to obtain the following objectives:

- Flexible materials were selected for the radiator due to their appealing features, including light weight and conformability [25].
- A loop structure was chosen and investigated in this work. This is because of its simplicity, making it well-suited for integration with flexible materials. Additionally, as mentioned earlier, loop antennas have been shown to be more sensitive than dipole antennas for ice detection applications [20].

Moreover, multiple concentric rings can be integrated within each other. This provides an area of coupling with the material under test. This coupling controls the effective capacitance of a parallel plate capacitor assembled from two rings separated by a dielectric in this case. The resulting capacitance also contributes to fine-tuning the antenna’s resonance and optimizing its impedance matching at 2.49 GHz [26,27].

(1) $$f_{r}=\frac{1}{2\pi \sqrt{L_{eff}C_{eff}}}$$

where $C_{eff}$ (F) is the total effective capacitance and $L_{eff}$ (H) is the total effective inductance.

The loop antenna also provides an omnidirectional radiation pattern. This pattern type is preferred in such applications in order to guarantee radiation from the sensor antennas to receivers in different directions.

- Resonance at around 2.49 GHz is within the 2.4–2.5 GHz Industrial, Scientific and Medical (ISM) band. This is an unlicensed band compatible with a wide range of wireless technologies, including sensor networks. The 2.45 GHz ISM band offers several advantages for early warning systems, such as moderate range and good penetration through foliage, rain and obstacles, which supports effective sensing in outdoor environments, particularly for frost, flood and wildfire detection. This band experiences lower atmospheric attenuation if compared with others at higher frequencies such as 5.6 GHz. This ensures a longer range of communication or more robust communication at shorter distances. Additionally, lower power consumption is usually associated with this band, which extends the lifetime of the battery-powered system. Also, many technologies have already been deployed utilizing this frequency band. Hence, real-time data collection and transmission can be supported for early warning systems utilizing the existing infrastructure [28].

The proposed antenna is supposed to detect ice or frost. Its performance is assessed through variations in the resonant frequency for the different materials under test. The change in the effective dielectric constant value ($\varepsilon_{reff}$) from one material to another causes the resonant frequency to alter as follows [29]:

(2) $$f_{r}=\frac{f_{0}}{\sqrt{\varepsilon_{reff}}}$$

where $f_{0}$ in Hz is the original resonant frequency without loading and $f_{r}$ in Hz is the resonant frequency for the antenna when loaded with the material under test.

Due to the different relative permittivity values for the different materials under test, such as ice and frost, the resonant frequency values vary differently in response to these materials. A solid layer on the antenna surface is used to model ice and frost while a layer of water heated up to 40 °C and 50 °C is used to resemble wildfire conditions, in correspondence with the work in [19]. The relative permittivity and conductivity of the materials investigated in this paper are summarized in Table 1 [1,19]. A temperature of 40 °C is considered sufficiently high to trigger the early warning system for wildfire [19].

It is worth indicating that the relative permittivity of materials is frequency-dependent in general. It decreases with frequency [30]. However, it can be considered approximately constant for the investigated materials listed in Table 1 over the relatively narrow frequency range around 2.45 GHz [31,32]. The data provided in Table 1 correspond to 2.45 GHz. On the other hand, the effective relative permittivity increases with the layer thickness [33]. This variation is utilized as a distinguishing feature to differentiate between distinct ice accumulation levels. This will be further explained in the following parts of the paper.

It is important to note that the proposed antenna should maintain a wide sensitivity range which is broad enough to include both the wildfire and frost scenarios despite the large difference between their corresponding relative permittivity values. This is obtained for the proposed design by controlling the effective capacitance, which varies with the effective permittivity. This will be explained in the following section.

## 3. Methodology and Methods

To assess the antenna performance and validate the concept, the following methodology is employed and followed:

- Design the antenna using simulation software with reference to theoretical basics. Computer Simulation Technology (CST) is used for this work [34]. Hexahedral meshes and a time-domain solver are used. A simulation accuracy of −40 dB is set, ensuring excellent convergence and stability and providing reliable and accurate results. In addition, open boundaries are selected to minimize reflections, which provides more accurate near and far field results and allows for modeling free space radiation accurately. The simulations are also run with a port impedance of a pure 50 Ohms resistance, ensuring compatibility with a real standard value for practical connectors.
- Simulate the antenna in free space under the following conditions:
  - Without any loading materials;
  - With a layer of ice-mimicking material;
  - With a layer of frost-mimicking material;
  - With water-equivalent material at 40 °C and 50 °C.

**Figure 3:**
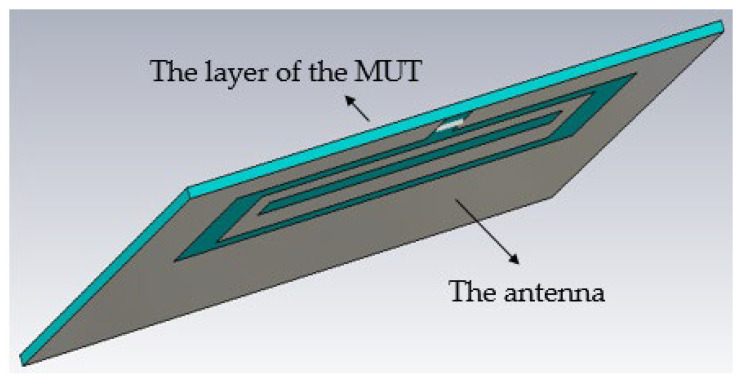
The proposed antenna with the material under test.

All the dielectric properties of the simulated layers are provided in Table 1. A layer thickness of 1 mm is employed at this stage. The antenna with the materials under test is shown in Figure 3.

The simulations at this stage assume the following:

- Free space conditions, excluding other parameters such as humidity and smoke;
- A steady material status during the overall duration of the simulation (i.e., it is not melted or starting to melt);
- The layer of the MUT is uniform;
- Fixed dielectric properties for materials over the entire simulation frequency range;
- A perfect conducting radiator.

- Study and analyze the simulated results. The key parameters (−10 dB matching, variations in the resonant frequency, radiation efficiency, gain, radiation pattern, communication distance) are evaluated at this stage. The effect of the ice layer thickness on the antenna performance is also evaluated at this stage.
- Validate the antenna performance. The antenna is fabricated at this stage and its reflection coefficient along with its resonant frequency are measured for the case of ice. The same conditions of simulations are kept through measurements. The measured results are compared with the simulated ones to validate the performance.

It is worth indicating that this method can be repeated across different antenna structures and designs.

Measurements were conducted inside a lab room in normal weather conditions in the middle of July.

## 4. Simulation Results

In this section, the simulation results for both the sensing and data transmission modes are presented and discussed. For the sensing mode, the resonant frequencies noted from the reflection coefficient results are mainly evaluated and studied, while the gain, radiation efficiency and radiation pattern are investigated for the data transmission mode.

### 4.1. Sensing

**Figure 4:**
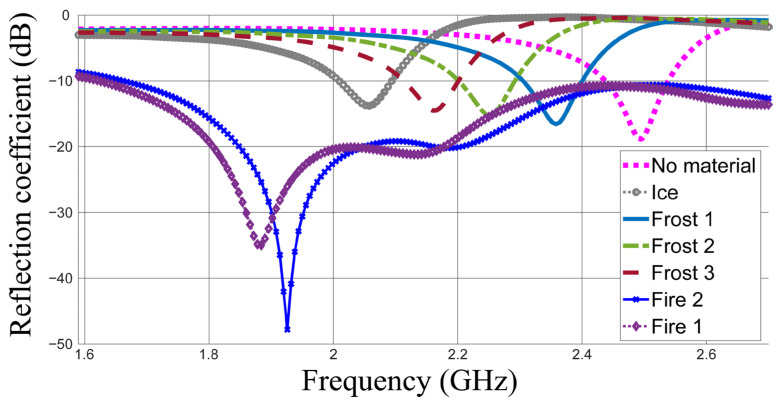
The simulated reflection coefficients in dB for the investigated materials being detected.

The main parameter of evaluation in the sensing case is the resonant frequency, which can be observed from the reflection coefficient. The results of investigations for the ice, frost and water layers at 40 °C and 50 °C are shown in Figure 4.

This figure indicates that the resonant frequency shifts down from 2.496 GHz in the case without loading to 2.358, 2.25, 2.166, 2.058, 1.962 and 1.878 GHz in the cases of frost 1, frost 2, frost 3, ice, fire 2 (50 °C) and fire 1 (40 °C), respectively. In general, the resonant frequency shifts down with the dielectric constant. The sensitivity level is indicated as Δ*f_r_* = 192 MHz per Δ*ε_r_* = 1 for the four materials of the same conductivity (frost 1, frost 2, frost 3 and ice). Howeverthe shift in the resonant frequency becomes smaller between the cases of water at 40 °C and 50 °C which is Δ*f_r_* = 84 MHz for a dielectric constant difference of Δ*ε_r_* = 4.

In real cases, ice is expected to accumulate on the antenna surface. Hence, its accumulation effect is evaluated. Thicker accumulated layers are expected to shift the resonant frequency down since the effective permittivity increases with thickness. This shift can be justified with reference to Equation (3) as follows:

(3) $$f_{loaded}=\frac{f_{unloaded}}{\sqrt{\varepsilon_{reff}}}$$

where $f_{loaded}$ in Hz is the resonant frequency after the antenna is loaded with the material under test, while$\;f_{unloaded}$ in Hz is the resonant frequency of the antenna before loading with ice, frost or water. $\varepsilon_{reff}$ is the relative effective permittivity, which increases with thickness for the same material under test.

**Figure 5:**
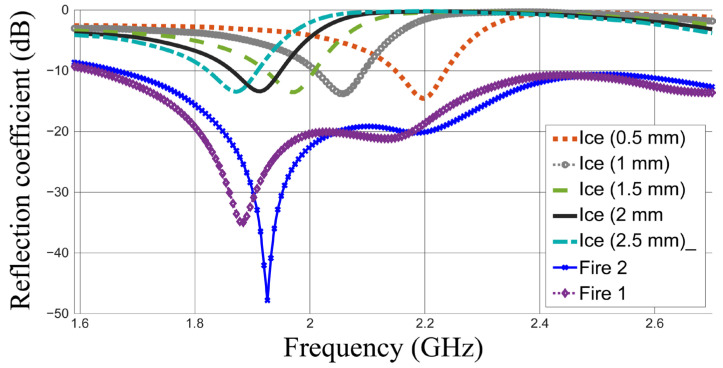
The simulated reflection coefficients in dB for the different thicknesses of ice compared to those of wildfire-resembling cases.

The simulated results are presented in Figure 5 and summarized in Table 2.

The results clearly indicate that an ice thickness of 2 mm shifts the resonant frequency down to approximately 1.914 GHz.

Initial studies reveal that ice layers thicker than 1.5 mm may shift the resonant frequency down below 2 GHz, which is the same frequency range noted for fire 1 and fire 2. Consequently, a dual-property detection approach is employed, utilizing an additional property or frequency band to determine the detected material. This concept has been introduced in our previous work presented in [1] where an alternative frequency band was used for material identification. However, the matching level is employed as an additional decision parameter in this work. This is because a much deeper matching level attributed to the high conductivity values in the cases of fire 1 and fire 2 will probably always be obtained [35]. This property can be reliable enough, as much deeper matching will always be obtained for fire 1 and fire 2 if compared with that in the ice cases.

The reflection coefficients for ice thicknesses of 0.5, 1, 1.5, 2 and 2.5 mm are shown in Figure 5. The resonant frequencies at 0.5 and 1 mm thicknesses of ice are always above 2 GHz. However, the resonant frequencies when the ice thickness increases become close to those of fire. Hence, the matching property can be used in addition to the property of resonant frequency. When the resonant frequency falls below 2 GHz and S_11_ < −20 dB, fire can be detected. It is worth noting that the temperature considered for early detection and alarm purposes in early warning systems is 40 °C.

The results are influenced by the thickness of the material under test. A thicker layer results in larger effective permittivity, which produces a larger frequency shift and thus a higher sensitivity level, defined as (Δ*f_r_*/Δ*ε_reff_*).

**Figure 6:**
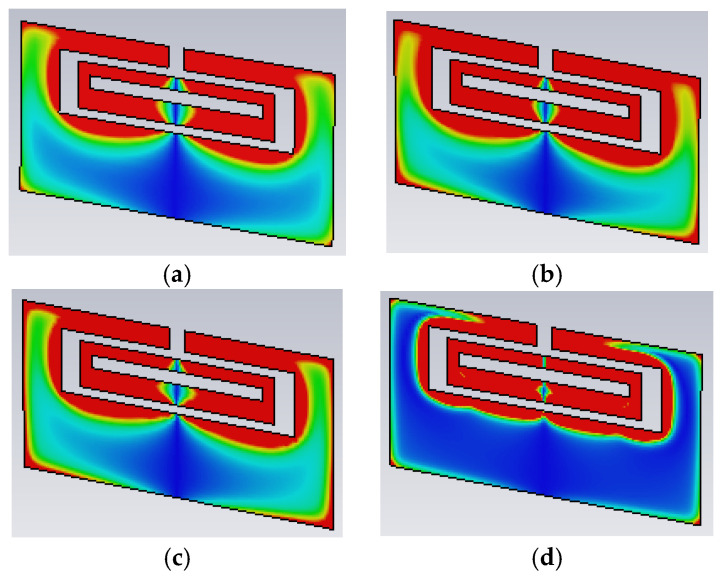
The near electric field of the proposed antenna: (**a**) without loading, (**b**) frost 1, (**c**) ice (1 mm), (**d**) fire 1. Colors indicate the field strength in ascending order are as follows: Dark blue (weakest), light blue, green, yellow and red (strongest).

The effect of the internal loop on the sensitivity is investigated by studying the near electric field of the proposed antenna without loading and with frost (1 mm), ice (1 mm) and water at 40 °C. The results are depicted in Figure 6.

This figure shows that the near electric field is the strongest at the inner ring and at the edges between the inner and outer loops. Stronger coupling with the material under test is introduced when the near electric field is increased, which enhances the sensitivity as desired.

### 4.2. Data Transmission

The gain and radiation efficiency of the proposed antenna for all the investigated cases are summarized in Table 2.

**Figure 7:**
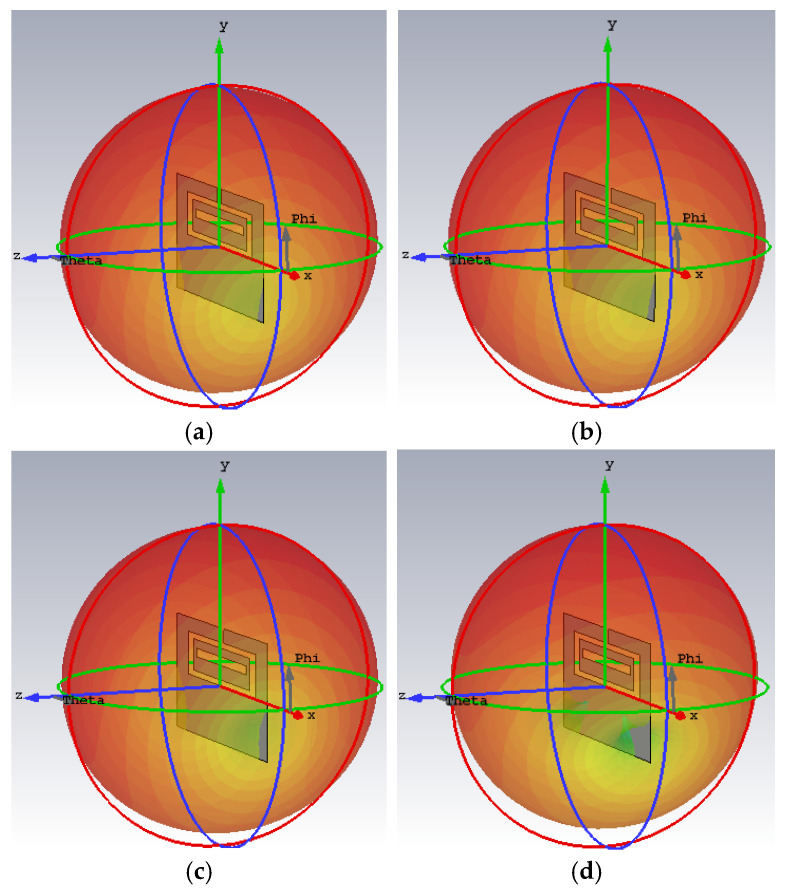
The maximum 3D gain radiation patterns of the proposed antennas at 2.45 GHz for the following cases: (**a**) without loading, (**b**) frost 1 (**c**) ice (1 mm),and (**d**) fire 1; red: Maximum, yellow: medium and green: minimum.

A radiation efficiency approaching one was obtained when the antenna was not loaded with any material. A maximum 3 D gain of 2.83 dBi was obtained in this case. The radiation efficiency and gain decrease after loading the antenna with different materials. This is expected, as extra losses are introduced when the antenna is loaded with lossy materials such as ice, frost and wildfire. Ice and frost have small losses represented by their small conductivities, which are close to each other. Hence, their corresponding gain and radiation efficiency values are comparable to each other and to the no-loading case. On the other hand, large conductivity values and losses are encountered in the cases of fire 1 and fire 2. This is reflected on decreasing the radiation efficiency by 15 times and gain by around 11 dBi. The radiation pattern is omnidirectional, with maximum radiation obtained at the same angle for all cases under investigation, as shown in Figure 7.

The link budget is calculated for all cases of detection. The link parameters are summarized in Table 3 [36], and the results are summarized in Table 4.

The distance can be calculated with reference to Equation (4).

(4) $$P_{r}=P_{in}+G_{tx}+G_{rx}-10nlog\left(d/d_{0}\right)-20\;log\;\left(4\pi df/\;c\right)-L_{C}-LM$$

$d_{0}$ is a reference distance which is considered to be 1 m, *f* is the resonant frequency and c is the velocity of electromagnetic waves in free space, which is equal to 3 × 10^8^ m/s.

Calculations are conducted for the frequencies obtained for each investigated MUT and their corresponding gain values. An input power of 20 dBm, receiver sensitivity of −89 dBm and receiving antenna gain of 14 dB [36] are assumed. A path loss exponent of three is used in the calculations considering a worst-outdoor-case scenario [24].

In general, a distance of longer than 300 m is always obtained for the antenna without loading and when loaded with ice or frost. This distance decreases to 157 m when loaded in the fire 1 case. This distance is sufficient to ensure reliable communication in real-world scenarios for the intended application.

The calculated distances are summarized in Table 4.

## 5. Validation and Discussion

**Figure 8:**
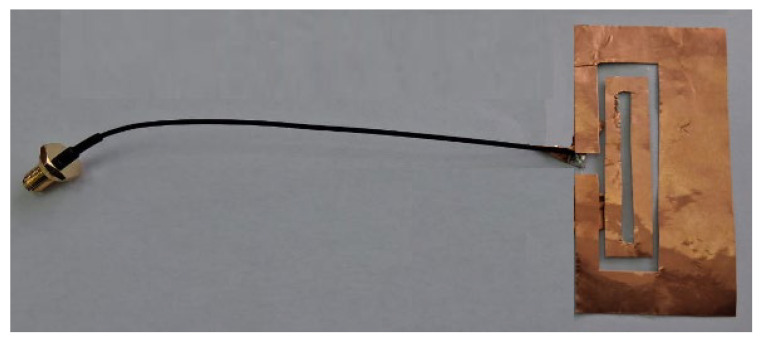
The fabricated antenna.

The antenna is made of a conducting copper foil of 0.035 mm thickness and an approximate conductivity of $5.96\times 10^{7}$ (S/m) [37,38]. It has a substrate layer of LDPE (Low-Density Polyethylene) which has an approximate relative permittivity ($\varepsilon_{r}$) of 2.2 and a loss tangent (tanδ) of 0.0004 [39] at 2.45 GHz. The substrate has a thickness of 0.1 mm. The antenna is fed using a coaxial cable, as shown in Figure 8.

**Figure 9:**
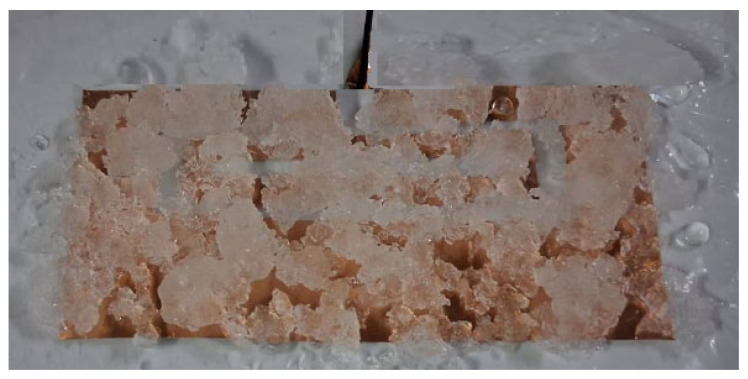
The proposed antenna loaded with ice.

The reflection coefficient (S_11_) was measured using an Anritsu ShockLine MS46322B two-port vector network analyzer (VNA), (Anritsu Company, Morgan Hill, CA, USA). The instrument has a frequency range from 100 MHz to 43.5 GHz, 130 microseconds per point sweep speed and better than 100 dB dynamic range to 43.5 GHz. It is operated in its standard S-parameter mode [40]. Calibration was performed using a SOLT (Short-Open-Load-Thru) kit; calibration standards were connected directly to the reference plane [41]. All measurements used a 50 Ω system impedance; cables with phase-stable 50 Ω coaxial connectors were used throughout. Additionally, care was taken to ensure that the connector did not contact any part of the material under test, avoiding unintended interactions in the measurements. A thin insulation layer is placed on the antenna surface between the antenna and ice. This is to isolate the ice’s effect from the coaxial cable and radiation from it. This layer is also made of LDPE and has a thickness of 0.1 mm. The proposed antenna loaded with ice for measurements is shown in Figure 9.

**Figure 10:**
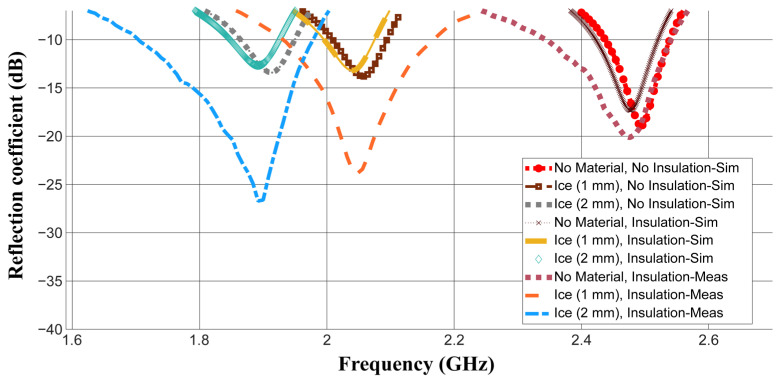
The measured and simulated reflection coefficient S_11_ (dB) of the fabricated antenna without loading and with ice layers of 1 and 2 mm.

The measured results for the antenna without loading and with ice are presented in Figure 10. Two ice thicknesses of about 1 and 2 mm are used.

The measured results are compared with the following:

- Simulated results without the insulation layer.
- Simulated results with the insulation layer, taking into account its actual thickness and dielectric properties provided above.

This is to provide an accurate comparison by modeling the real measurement case more precisely while investigating the insulation layer effect at the same time.

It can be seen from this figure that adding the insulation layer causes the resonant frequency to shift down. The simulated resonant frequencies are shifted down by 12, 17 and 21 MHz for the cases with no material, ice (1 mm) and ice (2 mm), respectively, after placing this layer on top of the antenna. This is expected because of the larger effective permittivity introduced after adding this layer, which has a relative permittivity of 2.2. The simulation results, including the insulation layer, demonstrate better agreement with the measurement results. Only 2, 7 and 1 MHz are noted as differences between the simulated and measured results with the insulation layer for the cases with no material, ice (1 mm) and ice (2 mm), respectively. This is likely due to the non-uniform ice layer used in the measurements. Nonetheless, this difference is very small. A deeper −10 dB bandwidth is obtained during measurements. This is probably due to the additional losses introduced by the impurities of ice and the insulation layer, which were not included in the simulations [42]. Such losses cause larger power absorption rather than reflection, and hence deeper −10 dB matching is obtained. However, this deep −10 dB level was still within a range sufficient to differentiate between the distinct materials (ice, frost and wildfire) and detect them accurately. This can be further explained by referring to Table 5, which summarizes different detection cases based on both simulated and measured resonant frequency ranges and matching levels.

Frost is always detected when a resonant frequency higher than 2.16 GHz is registered. An exception is noticed at 2.196 GHz in which an ice layer of a small thickness (0.5 mm) might be detected. However, this is acceptable, as frost can be considered a thin layer of ice [43]. In this case, precautions for frost damage can be taken when an alarm signal is received. Ice is always detected for a resonant frequency between 2 and 2.15 GHz. For frequencies below 2 GHz, ice can be detected at matching levels ranging from above −30 dB to −10 dB while wildfire can be detected at a deeper matching level, lower than −30 dB.

The proposed antenna is compared with other designs proposed in the literature for similar applications, with a summary provided in Table 6. The proposed antenna outperforms the design in [20] by obtaining a 7.72 dBi higher gain at an ice thickness of 1 mm only, although both designs are loops operating at the same frequency (2.45 GHz). This is attributed to the internal loop added to the proposed design. While the antennas in [1,19] exhibit higher gain values, this is expected as they operate at higher frequencies. The antenna in [5] achieved a slightly higher gain. However, a low sensitivity level was obtained for that design, with very small frequency shifts between the different tested materials.

The design in [44] utilized a double split-ring resonator to detect ice over a frequency range of 3.5 to 5 GHz. However, it was optimized and evaluated for sensing purposes rather than data transmission. The antenna in [45] was an RFID dipole optimized for ice detection at 915 MHz. Although it demonstrated good sensitivity, its received signal strength at 1 mm ice thickness was −59 dBm, which is 12 dBm lower than that of our design when used in the same system.

Regarding sensitivity, the proposed antenna is compared with others with documented data on frequency shifts for comparable materials and thicknesses. For example, the frequency shift obtained in [18] for ice was 36 MHz for a thickness of 1.5 cm. This is much smaller than the shift obtained for our design, which was 432 MHz for an ice thickness of 1 mm only. This ensures the design’s effectiveness in obtaining a high sensitivity level. The design in [1] obtained a frequency shift of 91 MHz only, which again indicates a low sensitivity level in comparison with the design proposed in this paper.

The contributions of this work can be summarized as follows:

- It involves a high sensitivity level combined with a relatively large gain compared to existing antennas of the same type operating at the same frequency.
- It proposes what is probably the first loop antenna proposed with a wide temperature detection range between 0 and 50 °C.
- It investigates and underscores the effect of multiple rings on the sensor loop antenna performance with the purpose of inspiring more optimized loop antenna structures for sensing applications in the future.
- It validates the robustness of flexible antennas for use in harsh environments, with applications in early warning systems and environmental monitoring.

## 6. Conclusions and Future Work

In this paper, a double rectangular ring loop antenna is designed and evaluated for the purposes of detecting ice, frost and water at temperatures of 40 °C and 50 °C, which resemble wildfire conditions. The antenna has shown a high level of sensitivity by responding with distinct resonant frequency shifts to different tested materials. A resonant frequency shift of 192 MHz was observed when the relative permittivity varied by only one for the four materials of the same conductivity (frost 1, frost 2, frost 3 and ice). This shift was 84 MHz between the cases of water at 40 °C and 50 °C. The matching level is also exploited in addition to the resonant frequency shift to distinguish the wildfire-resembling scenarios from ice layers thicker than 1.5 mm. The effect of the near electric field on the antenna sensitivity is investigated. The internal ring is found to increase the near-electric field strength and fine-tune the antenna performance. Additionally, calculations indicate that the antenna proposed in this work can send alarm signals over distances of 157 m for wildfire detection and 300 m for frost or ice detection. The antenna has shown robust performance with good matching between simulation and measurement results. It demonstrated robust performance when loaded with ice, maintaining consistent results in both simulations and measurements. The findings suggest that the multiple ring loop antenna is a promising candidate for sensing applications, offering a simple structure, excellent sensitivity, robust performance and relatively high gain compared to existing single loop antennas.

While the antenna has shown promising performance, further work should be conducted in the future. This includes the following:

- Further investigations on other contributing parameters such as smoke for wildfire detection and humidity may be needed in the future. This is to model the actual environment more accurately.
- Further work should investigate other loop and ring structures in addition to possible coupling techniques. For example, multiple split rings instead of a single ring may be investigated. The capabilities of the internal ring and split rings in strengthening the near electric field and boosting the sensitivity level may be studied.
- Measurements of the radiation efficiency, gain and radiation pattern should be conducted.
- Measurements over longer periods of time involving variations in the status of the material under test, which may include ice melting and temperature variations, should be conducted.
- Measurements after integration in a prototype sensing system should be conducted.
- Calculation and estimation of a more accurate link budget taking actual data rates and channel capacity into consideration should be conducted.

## Figures and Tables

**Table 1:** Relative permittivity and conductivity of the investigated materials under test at 2.45 GHz.

Material	Relative Permittivity	Conductivity (S/m)
ice	3.2	1 × 10^−5^
frost 1	1.5	
frost 2	2	
frost 3	2.5	1 × 10^−5^
High temperature 40 °C (fire 1)	71.7	1.59
High temperature 50 °C (fire 2)	67.7	1.59

**Table 2:** Maximum radiation efficiency and gain of the materials under test.

Case	Resonant Frequency	Gain in the Direction of Maximum Radiation (dBi)	Maximum Radiation Efficiency (%)
without material	2.49	2.83	98.7
ice (0.5 mm)	2.196	2.72	96.5
Ice (1 mm)	2.058	2.68	95.8
ice (1.5 mm)	1.968	2.672	95.96
ice (2 mm)	1.914	2.66	95.9
ice (2.5 mm)	1.872	2.652	95.8
frost 1	2.358	2.76	97.7
frost 2	2.25	2.714	97
frost 3	2.16	2.7	96.55
fire 1	1.884	−8.27	6.5
fire 2	1.926	−8.423	6.67%

**Table 3:** The investigated link parameters.

Parameter	Symbol	Value	Unit
frequency	f	2.49	GHz
		2.196	
		2.058	
		1.986	
		1.914	
		1.872	
		2.358	
		2.25	
		2.16	
		1.884	
		1.926	
input power	$P_{in}$	20	dBm
receiver sensitivity	$P_{r}$	−89	dBm
transmitter antenna gain	$G_{tx}$	2.83	dBi
		2.72	
		2.68	
		2.672	
		2.66	
		2.652	
		2.76	
		2.714	
		2.7	
		−8.27	
		−8.423	
receiver antenna gain	$G_{rx}$	14	dBi
link margin	LM	10	dB
reference distance	$d_{0}$	1	m
path loss exponent	n	3	---
cable loss	$L_{C}$	2.76	dB

**Table 4:** Distance of communication of the investigated link.

Case	Distance (m)
without	315.355
ice—0.5 mm	338.844
ice—1 mm	353.9786
ice—1.5 mm	364.754
ice—2 mm	370.936
ice—2.5 mm	375.84
frost 1	323.594
frost 2	334.426
frost 3	343.8745
fire 1	157.76
fire 2	162.0317

**Table 5:** The detection cases based on frequency range and matching level.

Frequency Range in GHz	Matching Level (S_11_) in dB	Case
>2.16	−20 to −10	frost
2–2.15	>−30 to −10	ice
<2	>−30 to −10	ice
<2	<−30	wildfire

**Table 6:** Comparison with previous work.

Ref.	Resonant Frequency (GHz)	Sensing Technique	Antenna Type	Gain (dBi)	Targeted Application
[1]	5.6	resonant frequency	cross-slotted patch	ice: 3.52 frost: 4.052	ice, frost and water detection
[5]	2.45 (data transmission) 4.9 (sensing)	resonant frequency	modified monopole with a T-shape patch antenna	3.5 not provided	ice and water detection
[18]	2.45	resonant frequency (shifts and amplitude)	T-slotted patch antenna	ice (4.3) water (4.9)	frost, ice and water detection
[19]	5.4	resonant frequency	substrate-integrated waveguide	~5.2	frost and wildfire detection
[20]	2.4	received signal strength	loop	−5: ice (1 mm)	ice thickness measurements
			dipole	−3: ice (1 mm)	
[44]	3.5	resonant amplitude and transmission coefficient	split-ring resonator	not provided	ice detection
[45]	915	received signal strength	dipole	not provided	ice detection
This work	2.45	resonant frequency and matching level	flexible loop	frost: 2.76 ice: 2.72 wildfire: −8.27	frost, ice and wildfire detection

## Data Availability

The data presented in this study are available on request from the corresponding author.
